# Elective surgeries during and after the COVID-19 pandemic: Case burden and physician shortage concerns

**DOI:** 10.1016/j.amsu.2022.104395

**Published:** 2022-08-19

**Authors:** Aashna Mehta, Wireko Andrew Awuah, Jyi Cheng Ng, Mrinmoy Kundu, Rohan Yarlagadda, Meghdeep Sen, Esther Patience Nansubuga, Toufik Abdul-Rahman, Mohammad Mehedi Hasan

**Affiliations:** a University of Debrecen-Faculty of Medicine, Debrecen, Hungary; b Sumy State University, Sumy, Ukraine; c Faculty of Medicine and Health Sciences, University of Putra Malaysia, Serdang, Malaysia; d Institute of Medical Sciences and SUM Hospital, Bhubaneswar, India; e Rowan University School of Osteopathic Medicine, Stratford, NJ, USA; f American University of Antigua, St John's, Antigua and Barbuda; g Leeds Medical School, University of Leeds, UK; h Department of Biochemistry and Molecular Biology, Faculty of Life Science, Mawlana Bhashani Science and Technology University, Tangail, Bangladesh

**Keywords:** Surgery, Surgical care, COVID-19, Healthcare, Physician shortage

## Abstract

The COVID-19 pandemic had a significant impact on several aspects of global healthcare systems, particularly surgical services. New guidelines, resource scarcity, and an ever-increasing demand for care have posed challenges to healthcare professionals, resulting in the cancellation of many surgeries, with short and long-term consequences for surgical care and patient outcomes. As the pandemic subsides and the healthcare system attempts to reestablish a sense of normalcy, surgical recommendations and advisories will shift. These changes, combined with a growing case backlog (postponed surgeries + regularly scheduled surgeries) and a physician shortage, can have serious consequences for physician health and, as a result, surgical care. Several initiatives are already being implemented by governments to ensure a smooth transition as surgeries resume. Newer and more efficient steps aimed at providing adequate surgical care while preventing physician burnout, on the other hand, necessitate a collaborative effort from governments, national medical boards, institutions, and healthcare professionals. This perspective aims to highlight alterations in surgical recommendations over the course of the pandemic and how these changes continue to influence surgical care and patient outcomes as the pandemic begins to soften its grip.

## Highlights

• The COVID-19 pandemic had a significant impact on several aspects of surgical care.
• New surgical recommendations amidst an ever-increasing demand for care pose, short and long-term consequences for surgical care and patient outcomes.
• As the pandemic subsides, these changes, combined with a growing case backlog and a physician shortage, can have serious consequences for physician health and, as a result, surgical care.

## 1 Introduction

**Fig. 1:**
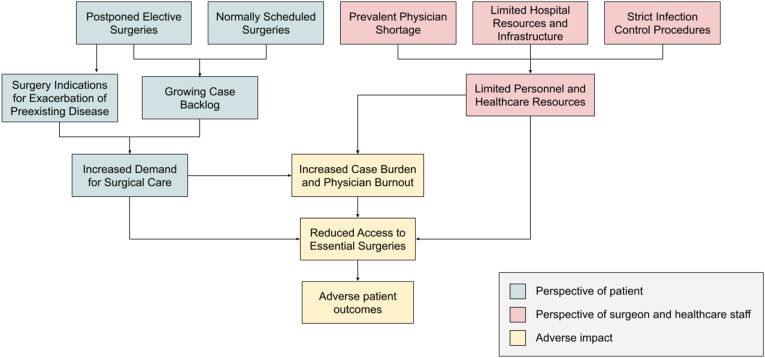
Schematic Representation of different aspects of surgical care impacted by COVID-19-associated surgical guidelines.

Described as a pandemic by the World Health Organization in March 2020, COVID-19 disease inadvertently has put enormous strain on the healthcare system, affecting both patient care and physicians especially in countries with vulnerable health systems [1,2]. With a significant increase in demand for medical help amidst high costs for protective equipment and shortage of supplies, the pandemic has intricately highlighted the socioeconomic and ergonomic vulnerabilities of the healthcare industry [2]. In response, several public health policies were formulated, governments issued numerous recommendations to mitigate spread [3]. Recommendations encompassed various aspects of healthcare including prevention through use of personal protective equipment (PPE such as KN95 mask, goggles, and gowns), social distancing, contact tracing, and regular testing for healthcare workers; management guidelines (supportive care); investment and resource allocation on healthcare infrastructure [2,3]. Like other medical specialties, surgeons were required to modify their expectations and responsibilities in order to ensure resource redistribution and meet care demands. The prioritization of specific surgeries, as well as the need for immediate but appropriate guidelines, had become critical. While most surgeries are necessary, interpreting the meaning of “elective surgeries” and ensuring patient safety was no easy task. Governments issued recommendations on elective procedures, but with new state orders and societal recommendations, surgeons were left with little to no guidance, resulting in a general decline in physician and patient well-being [4]. During the early stages of the pandemic, the American College of Surgeons (ACS) advised postponing non-urgent surgeries. Surgeries were classified into different tiers based on their urgency, ranging from tier 1a surgeries like carpal tunnel release to tier 3b surgeries like high acuity surgeries [5]. Also, millions of procedures were canceled, resulting in a variety of potential long-term and short-term effects on patient care, as indicated in Fig. 1. The long-term effects included the risk of uncertain loss of function and adverse prognosis as a collateral effect of the pandemic [6]. While short-term effects included deterioration in patients' conditions, increased disability, and reduced ability to work [7]. Sims et al. reported that the cancellation of elective surgeries had a more severe impact on women and black patients, highlighting the importance to address healthcare disparity to ensure equitable care [8].

Amidst efforts to maximize patient care, physician wellness was perhaps another pivotal aspect brought into light during the pandemic as it influenced productivity and care provision. According to a study conducted by Farr et al., primary care and surgery residents reported a significant reduction in dating frequency and socialization due to the pandemic [9]. It is obvious that the pandemic had a significant impact on almost every aspect of healthcare delivery and surgical care. As the world adjusts to the current pandemic in order to re-establish a new sense of normalcy, it is critical to address the pandemic's impact on surgical care. This viewpoint emphasizes the pandemic's impact on surgical recommendations, with a focus on surgical care and physician wellness.

### 1.1 The general impacts of covid-19 on elective surgeries

The COVIDSurg Collaborative study estimated that during a 12-week period of peak COVID-19 disruption, 28.4 million surgeries would be canceled or postponed worldwide [7]. However, this estimate could be far from reality as the pandemic is still around after 2 years from the study. The best estimate of the global 12-week cancellation rates for elective surgeries was 72·3%, which is consistent with the finding of another systematic review, where 74.4% of the articles recommended postponing all, or at least selected, elective surgeries [7,10]. A study predicted that the number of canceled surgical procedures in England and Wales during the COVID-19 pandemic is at least 2.4 million by the end of 2021, which represents more than 6 months of normal surgical activity [11]. Meanwhile in Brazil, the elective surgical backlog has surpassed 900,000 cases by December 2020 [12]. According to the COVIDSurg Collaborative study, 37·7% of cancer surgeries were affected [7]. Other than oncological surgeries, other specialties have also been affected. In the United States, up to 71.8% of live donor kidney transplants were completely suspended while over two-thirds of live donor liver transplants were completely suspended [13]. More than a 50% drop in cardiac surgical volume was observed at 2 the busiest cardiac surgical programs in Maryland after full restrictions were implemented by the ACS in response to the pandemic [14]. Another study projected a cumulative backlog of more than 1 million joint and spinal surgical cases in the United States by May 2022 [15]. In urology, there was an 89.6% reduction in elective surgeries during April 2020 in Brazil [16]. A multidisciplinary oncologic review of time to surgery showed delaying surgery for more than one month negatively impacts survival in breast cancer, T1 pancreatic cancer, Stage I melanoma, ovarian cancer, and pediatric osteosarcoma [17]. Decreased survival was also associated with surgical delays of >3 months in patients with hepatocellular cancer and 40 days in colorectal cancer [18]. A 3-month delay to surgery across all stage 1–3 cancers has shown to cause more than 4700 attributable deaths per year in England while a 6-month delay could cause more than 10000 attributable deaths per year [19]. A French study observed a mean 43-days postponement period for scheduled pancreatic adenocarcinoma surgeries, with a major switch from upfront surgery to waiting for neoadjuvant chemotherapy in patients with the respectable disease [20]. More frequent metastasis discoveries during surgery (13% compared to 2.2% before the pandemic) were reported [19]. Delaying elective surgeries even for benign conditions could be risky, causing a significant impact on patients' health [20]. In a Spanish study among pending cases for elective cardiac invasive procedures during the pandemic, the mortality at 45 days due to cancellation was 1.7%, whereas 8.3% of the patients had to undergo an urgent procedure due to clinical destabilization [21]. Besides, surgical delays have a risk of progressing into a more advanced disease, which often requires more intense and costly treatment [22]. Orthopedic surgeries were among the biggest proportions of elective surgeries being delayed. A study has found that 1 in 4 patients reported substantial physical and/or mental impact due to the cancellation of elective total joint arthroplasty during the pandemic [23]. Other than the discomfort and inconvenience of rescheduling surgery, there is a risk of muscle wasting due to immobility, decreased quality of life, and depression with increased susceptibility to substance use disorders [24,25]. Psychological burden including severe restrictions in private life has been reported among patients who had to postpone sexual or reproductive health surgeries [26]. As the pandemic continues, the consequences reported are likely the tip of the iceberg, thus more research can uncover the true impact, especially in low-middle income countries.

### 1.2 Expected changes in surgery recommendations post-pandemic and its effect on patient caseload

As most elective surgeries were postponed during the COVID-19 pandemic, the healthcare system must prepare for a notable demand of surgical care as the pandemic subsides. According to the COVIDSurg Collaborative study, it would take a median of 45 weeks to clear the backlog of surgeries during 12 weeks of peak pandemic disruption if the countries increased their normal surgical volume by 20% after the pandemic [9]. At 2 busiest cardiac surgical programs in Maryland, the surgical backlog would require a monthly operating volume of 216%–263% of baseline to clear, or 1–8 months based on various post-pandemic operational capacities [14]. To clear the backlog of delayed total knee arthroplasty surgeries, it would take the US health system 16 months [24]. Financial-wise, a study has found that the cost of clearing the post-pandemic waiting list (more than 2.3 million overdue or canceled surgical procedures) in the NHS in England is €5.3 (3.1–8.0) billion, excluding additional costs of delivering surgical services under strict infection control procedures (including PPE, preoperative screening and extra bed-days in hospital), which may cost more than €500 million [2,6]. This could lead to catastrophic expenditure, particularly in low–middle-income countries, which further increases the financial burden of surgery. As the COVID-19 pandemic slowly subsides, there is a need for new evidence-based guidelines which can provide cost-effective solutions to prevent disease transmission and cross infection, without excessively increasing treatment costs [27]. Multiple organizations such as ACS, the American Society of Anesthesiologists (ASA), the Association of periOperative Registered Nurses (AORN), the American Hospital Association (AHA), and the Royal College of Surgeons have provided multiple guidelines for resuming elective surgeries [28,29]. Recommendations include institutions accounting for the possibility of repeat waves of SARS-CoV-2 infection when planning post-pandemic surgical recovery to best explore strategies to safely resume elective surgeries and slowly increase the case volume. Besides, a prioritization strategy with a standardized scoring system should be established to help with re-ordering of previously canceled and postponed elective surgeries, as well as specialties prioritization (cancer, organ transplants, cardiac, trauma) [[28], [29], [30], [31]]. Other than that, institutions should ensure an appropriate number of intensive care units (ICU) and non-ICU beds, personal protective equipment (PPE), medical-surgical supplies, and trained staff appropriate to the number of elective surgeries to be performed without compromising the standard of care [28,32]. COVID-19 testing policies are likely to be implemented to protect staff and patient safety [28]. As the COVID-19 pandemic hits, telemedicine has emerged quickly as it allows physicians to provide care with safe distancing. Although there are still issues to address, telemedicine is likely to play a role in the preoperative and postoperative phases of care beyond the pandemic [33].

### 1.3 Impact of case burden on patient care and physician well being

Physician well-being is the cornerstone of every well-functioning health system because it improves the quality of patient care, increases patient satisfaction, and decreases medical errors. In this time of a global pandemic healthcare workers are under a huge workload. This overwhelming burden of cases could lead to caregiver burnout. So far, the burnout syndrome, involving mental exhaustion, loss of motivation, depersonalization or cynicism, and reduced professional efficiency has been empirically described and applicable to physicians [34,35]. The major causes of burnout are long work hours, sleep deprivation, fatigue, exhaustion, and the risk of infection [36]. Furthermore, unique pandemic-related pressures further deteriorate physician well-being [6,31]. A systematic review and meta-analysis of 13 studies of mental health during the COVID-19 pandemic published up to April 17, 2020, of which 12 were from China and 1 from Singapore, reported a pooled prevalence of 23.2% for anxiety, 22.8% for depression, and 38.9% for insomnia [37]. In another study on the mental health of healthcare professionals in China, they found intense psychological experiences, traumatization, and various mental health disorders among healthcare workers during the pandemic [38]. Because of these issues many physicians have expressed a desire to quit medicine [39], this could negatively impact the quality-of-care provision in a prevalent physician shortage with an increased case burden. This could have serious consequences on patient care and could strain the healthcare system further [40].

### 1.4 The issue of physician shortage, with special emphasis on surgical specialties

Surgical residents, fellows, and early-career surgeons face unique challenges during this global pandemic. During the 2003 SARS outbreak in southeast Asia, residents reported that their education was compromised as teaching sessions, grand rounds and elective surgeries were canceled [41]. Similar policies are adopted in this pandemic, reducing resident-educator contact with resident didactics like grand rounds either canceled or made virtual [42]. Clinical volume is also affected, as most of the elective surgeries and outpatient clinics have been postponed. Moral distress is a condition where the individual knows what is morally right but is unable to do so because of institutional or other constraints [43]. Residents are prone to moral distress because they are often responsible for implementing care plans, and they do not have the authority of developing [44,45]. The pandemic amplifies a number of these challenges [41]. Studies from various humanitarian organizations have reported that moral distress increases dropout rates and sick leave among disaster responders, this trend is also seen in the case of medical residents. Physician shortage has become rampant worldwide, including in developed countries [46]. The US is already facing a growing shortage of physicians and COVID19 has exacerbated this. It is estimated that from 2019 to 2034 there will be a shortage of 21,000 to 77,100 specialty physicians [47]. The NRMP data displays 39,205 residency spots, among which only 5538 are surgical spots (categorical + preliminary), highly unproportionally to the expected healthcare demand [48]. To address the shortage, countries have implemented independent approaches. The Italian government allowed newly graduated medical doctors or last-years residents to work in the COVID workforce to face the shortage of doctors [49]. In the US, several med schools allowed final-year students to graduate early or return to the hospital [50]. Other various institutes have shifted surgical specialists (Surgeons, Anesthesiologists, and Dermatologists) to work in COVID-19 units [47].

### 1.5 Efforts and recommendations

As we approach our third year into the pandemic, the world has begun the process of returning to a more familiar semblance of life as it was prior to COVID-19. With the pandemic softening its grip on various industries, elective surgeries need not be delayed. It is important to note that elective surgeries, as per the American College of Surgeons, are “essential surgeries” [8,51,52]. Many nonemergent surgeries, such as for cancer care, may face dire consequences if delayed considerably. One paper predicted a rise in deaths by nearly 10,000 from colon cancer due to a delay of over 4 months [53]. Additionally, any delay of an essential surgery poses an increased risk of acute exacerbations, infections, and surgical complications.

A Google Trends analysis looked to identify shifting public perspectives on the desire to pursue elective surgeries. While search volume has yet to return to pre-pandemic levels, the investigator's analysis indicates a steady rise in public interest that foreshadows an increased demand for previously deferred care [54]. The question therefore becomes how to facilitate this incoming demand. Firstly, Sastre et al. (Jan 2022) published a study suggesting elective surgeries could be safely resumed without concern for increased COVID-19 risk even in high incidence regions [55]. An option is to develop helpful screening tools to stratify patients on the waiting list by priority due to health status [56]. Another study showed that strict adherence to protocols allowed for elective surgeries to be completed with fewer complications, readmissions and mortality compared to 2019 [57]. Amutharasan et al. (2022) recommended the importance of self-isolation prior to surgery, the designation of a surgical team member for COVID-19 swabs, and the reinstatement of in-person pre-operative assessments to help guide patient trust and care [58].

Based on these findings, our recommendation would begin by urging the resumption of elective surgeries. Considering that most surgical services would have significant waiting lists, these groups should perform a priority stratification of their waiting list based on risk factors such as the urgency of the procedure, patient health status, and duration of delay since the initial surgical consult. As mentioned earlier, studies have indicated the safety of surgeries despite COVID-19, with no documented increased risk of its infection post-operation. Yet, we would suggest appointing a few members of the patient team (i.e., comprised of nurses, scrub techs, office administrators, etc.) who would be responsible for COVID-19 nasal swabs on patients' pre-operation, confirming negative results prior to arrival, and ensuring strict adherence to sanitation protocols pre-and post-operation. Finally, we suggest providing pamphlets or info sheets informing patients of safe post-surgery practices to avoid COVID-19 infection (i.e., isolation, continued mask use, sanitation, etc.). Further research and policy on the above recommendations will be vital to meet surgical care objectives while avoiding physician burnout.

## 2 Conclusion and outlook

The pandemic has been detrimental to surgical care. The extent of the impact unravels daily with increases in costs and resource demands due to delayed surgical procedures existing alongside normally scheduled procedures. Lengthy waiting lists shall prevail, but stratification of patients and procedures may improve care provision. Furthermore, the pandemic may encourage research on health policies and guidelines to cope with increasing post-pandemic surgical demands.

## Ethical approval

NA.

## Sources of funding

NA.

## Author contribution

Contribution to the work's conception and design: All authors under the supervision of Aashna Mehta, Wireko Andrew Awuah, and Mohammad Mehedi Hasan. All authors worked together to draft the work and revise it critically, with the help of Aashna Mehta, Wireko Andrew Awuah, and Mohammad Mehedi Hasan. The final version of the manuscript was read and approved by all of the authors.

## Registration of research studies

1. Name of the registry: NA
2. Unique Identifying number or registration ID: NA
3. Hyperlink to your specific registration (must be publicly accessible and will be checked): NA

## Guarantor

Mohammad Mehedi Hasan Department of Biochemistry and Molecular Biology, Faculty of Life Science, Mawlana Bhashani Science and Technology University, Tangail, 1902, Bangladesh. Email: mehedi.bmb.mbstu@gmail.com.

## Consent

NA.

## Data availability statement

No data available.

## Conflicts of interest

NA.
